# Plasma lipoprotein (a) and tissue plasminogen activator are associated with increased risk of atherosclerotic cardiovascular disease

**DOI:** 10.1016/j.heliyon.2022.e09836

**Published:** 2022-06-30

**Authors:** Fadia Mayyas, Eman Bani Omar

**Affiliations:** Department of Clinical Pharmacy, Faculty of Pharmacy, Jordan University of Science and Technology, Irbid, Jordan

**Keywords:** Atherosclerotic cardiovascular disease, Coronary artery disease, Lipoprotein a, Tissue plasminogen activator, Low-density lipoprotein

## Abstract

Atherosclerotic cardiovascular disease (ASCVD) is the most common cause of mortality. Lipoprotein a (Lp(a)) is a low-density lipoprotein (LDL)-like particle with a similar structure to tissue plasminogen activator (t-PA) and it competes with plasminogen for its binding site leading to reduced fibrinolysis. The aim of this study was to assess association of Lp(a) and t-PA levels with risk of ASCVD and whether they are dependent on LDL levels. Patients who presented to the catheterization lab for assessment of coronary artery disease were included and stratified by their risk of ASCVD into low, moderate, high, and very high risk. Plasma levels of Lp(a) and t-PA levels were measured before catheterization. Consecutive patients (n = 362) were included. The mean age±sem was 52.28 ± 0.60 years. Plasma Lp(a) and t-PA levels were higher in very-high and high-risk patients relative to low-risk patients. Serum levels of triglyceride and high-density lipoprotein but not LDL were correlated with risk of ASCVD. Plasma Lp(a) and t-PA were not correlated or modified with LDL level. Plasma Lp(a) and t-PA levels were higher in patients undergoing coronary revascularization relative to patients having no intervention. Plasma t-PA level was higher in patients presented with myocardial infarction compared to those with angina. Multivariate analysis documented independent association of Lp(a) and t-PA with ASCVD risk. Plasma Lp(a) and t-PA levels are associated with increased ASCVDASCVD risk independent of LDL and could be used as predictors of atherosclerosis risk and in selecting patients who may benefit from coronary revascularization.

## 1 Introduction

Atherosclerotic cardiovascular disease (ASCVD) is the most common cause of morbidity and mortality [1, 2]. Among deaths from cardiovascular diseases (CVD), coronary artery disease (CAD) is the first leading cause of death worldwide, followed by cerebrovascular disease [1, 2].

The low-density lipoprotein (LDL) is the most atherogenic lipid particle and is considered an independent risk factor of CAD [3, 4]. Another lipoprotein particle that resembles LDL is lipoprotein a (Lp(a)), an LDL particle with an apolipoprotein-A (apo-A) moiety covalently bound to its apolipoprotein-B (apo-B) component [5]. The structure of Lp(a) is similar to the plasminogen and tissue plasminogen activator (t-PA), and it competes with plasminogen for its binding site [6, 7] leading to reduced fibrinolysis.

The t-PA is a serine protease enzyme found on endothelial cells of blood vessels lining. It converts plasminogen to its active form, plasmin, which subsequently dissolves the blood clot and causes fibrinolysis [8]. Because Lp(a) inhibits the function of plasminogen activator inhibitor-1 (PAI-1), it results in enhanced coagulation. Levels of plasma Lp(a) are genetically inherited and relatively stable regardless of dietary habits or risk factors, making it ideal gene to predict future risk of CVDs [9]. Interestingly, elevated plasma Lp(a) level predicts future coronary heart disease events [10, 11]. Plasma Lp(a) concentration is associated with increased risk of stroke [12]. A prospective cohort study confirmed the association of high Lp(a) levels with poor prognosis after percutaneous coronary artery intervention (PCI) in stable CAD patients, suggesting the usefulness of Lp(a) measurements in predicting poor clinical prognosis before selective PCI [12]. Thus, plasma Lp(a) screening in patients with CVD risk has been recommended [13, 14]. Interestingly, elevated Lp(a) level was associated with acute MI in patients with normal LDL levels [15], clearly emphasizing the significance of this factor in patients with ischemia. However, it is unclear if Lp(a) level is elevated in moderate to high-risk patients without MI or established ASCVD.

Similar to Lp(a), an elevated baseline level of t-PA was associated with three to four times higher risk of MI and stroke compared to healthy subjects [16]. Intriguingly, studies showed a statistically significant association between circulating t-PA and subsequent CAD [17], and CVD events [18]. The association of high t-PA with CAD may suggest its association with endothelial injury and plaque rupture [17]. In Jordanian population, it is unknown if plasma Lp(a) and t-PA can predict ASCVD risk status in moderate to high risk patients without underlying CAD compared to those with CAD and whether they are correlated with LDL concentrations. In addition, it is unclear if plasma Lp(a) and t-PA could predict the severity of CAD and need for coronary revascularization. Previously, we have shown that plasma levels of inflammatory and oxidant factors are associated with ASCVD risk regardless of LDL levels [19].

In this study, we tested plasma Lp(a) and t-PA levels in Jordanian patients with low to very high risk of ASCVD and their usefulness in predicting ASCVD risk status and need for coronary intervention.

## 2 Methods

### 2.1 Study design

This is a cross-sectional study that was conducted at King Abdullah University Hospital (KAUH) from April 2020 to March 2021. The study was approved by the Institutional Review Board (IRB) of Jordan University of Science and Technology and KAUH. All procedures were performed in accordance with the ethical standards of the Helsinki Declaration. A written informed consent was obtained from all included adult patients explaining the protocol and the potential hazard and benefits of the study.

### 2.2 Inclusion and exclusion criteria

Inclusion criteria included consecutive patients (>18 years old) presenting to the catheterization lab with angina that requires clinical assessment. Coronary artery disease (CAD) was established as ≥50% stenosis in one or more of the major coronaries.

Patients were classified according to the national lipid association (NLA) for ASCVD risk assessment into [20]:

1. Very high risk: patients who have established ASCVD (CAD, MI, stroke, peripheral artery disease (PAD), and any type of atherosclerosis), DM with greater or equal to two ASCVD risk factors or evidence of end-organ damage.
2. High risk: patients who have three or more ASCVD risk factors, DM with 0–1 ASCVD risk factor and no evidence of end-organ damage, chronic kidney disease (CKD) stage 3B or 4, quantitative risk score reaches the high-risk threshold (20%) by the pooled cohort equation, or LDL ≥190 mg/dl.
3. Moderate risk: patients who have two major ASCVD risk factors.
4. Low risk: patients who have 0–1 major ASCVD risk factors.

According to the American Heart Association/American College of Cardiology (AHA/ACC), major risk factors for ASCVD that were considered are: age (male ≥45 years, female ≥55 years), family history of early coronary heart disease (first-degree relative at age <55 in men and <65 in women), current cigarette smoking, hypertension (BP ≥ 140/90 mm Hg), and low HDL (<40 and <50 mg/dL) for men and women, respectively.

Few patients with low to moderate ASCVD risk were recruited from the cardiology outpatient clinic.

Exclusion criteria were patients with an inflammatory disease or infection, recent surgery or trauma, and renal dysfunction (creatinine >1.5 mg/dL). Demographic, clinical, and laboratory data were obtained by query of the patients' data files at the cardiology clinic and catheterization lab of KAUH. A specific data sheet was extracted for each patient describing patient characteristics, including use of medications. Plasma lipid contents, complete blood count, and other routine functional parameters for included patients were obtained from patients' electronic data files.

### 2.3 Blood collection

Freshly blood samples were withdrawn from patients from the femoral vein before intake of oral or intravenous medications. Few samples were also obtained at the hospital laboratory. Blood samples were transferred on ice and centrifuged at 2500 rpm for 10 min to separate plasma. Plasma was stored at -80 Cº until analysis. Plasma biomarkers content was assessed using commercially available ELISA kits. All samples were analyzed at one time after study completion. The analyzer was blinded to patients' groups.

### 2.4 Measurments of plasma lipoprotein (a)levels

Levels of plasma Lp(a) were evaluated using an ELISA kit according to manufacturer's instructions (Human lipoprotein a ELISA kit, Novus biologicals, Colorado, USA). The microplate was coated with an antibody specific for Lp(a). Human Lp(a) samples and standards (50 μL) were added to each well. The plate was incubated at room temperature for 2 h and was washed 5 times with 200 μL of washing buffer per well. A 50 μL of biotinylated human Lp(a) antibody was added to each well, and the plate was incubated for 1 h at room temperature. After washing, 50 μL of streptavidin peroxidase conjugate was added to each well, and the plate was incubated for 30 min at room temperature. Then, a 50 μL of chromogen substrate was added followed by 50 μL of stop solution. The absorbance of each well was determined at 450 nm using a spectrophotometer microplate reader.

### 2.5 Measurments of plasma tissue plasminogen activator (t-PA) levels

The levels of plasma t-PA were measured using an ELISA kit (Human t-Plasminogen Activator, R&D Systems, Minnesota, USA). The diluted capture antibody was added into a 96-well microplate and the plate was incubated overnight at room temperature. The wells were washed three times, blocked and incubated at room temperature for 1 h. Following washing, a 100 μL of sample or standard was added and incubated for 2 h at room temperature. After washing, 100 μL of the detection antibody was added and incubated for 2 h at room temperature. After washing, 100 μL of the working solution of streptavidin-horseradish peroxidase was added to each well and incubated for 20 min at room temperature. Then, a 100 μL of substrate solution was added to each well followed by the addition of 50 μL of the stop solution. The optical density of each well was determined at 450 nm using spectrophotometer microplate reader.

### 2.6 Statistical analysis

Data are expressed as mean ± standard error of the mean for continuous data and frequencies for categorical data. Normally distributed variables were analyzed using one-way analysis of variance, whereas non-normally distributed data were analysed using Kruskal-Wallis tests. Dunn's and Tukey post hoc tests were used for multiple comparisons. Chi-square tests were used to compare frequencies between groups. Spearman correlation was used to study the correlation of non-continuous data. Univariate analysis and figures were performed using GraphPad Prism 9. Step-wise multivariate analysis was used to adjust for covariates and possible confounders using JMP 13 software (SAS institute). Statistical significance was set at *p*-value < 0.05.

## 3 Results

### 3.1 Patients characteristics

Plasma samples from 362 consecutive patients were used for analysis of Lp(a) and t-PA levels and other lab measurements. Table 1 shows study groups classified based on their 10-year primary risk of ASCVD. Study groups included: 1) Low risk patients for ASCVD (Low, N = 50); 2) Moderate risk patients for ASCVD (Moderate, N = 54); 3) High risk patients for ASCVD (High, N = 37); and 4) Very high risk patients (Very High, N = 221). The mean age of the patients was 52.28 ± 0.60 years (mean ± SEM), and 246 patients were males (68%).

**Table 1:** Patients' characteristics.

	Low N = 50	Moderate N = 54	High N = 37	V. High N = 221	P value
Age	41.31 ± 1.48	48.83 ± 1.42∗	53.45 ± 1.70∗	55.35 ± 0.69∗	<0.0001∗
Male gender	22 (44.0)	33 (61.11)	22 (59.46)	169 (76.74)	<0.0001∗
BMI	28.76 ± 0.83	28.69 ± 0.79	28.99 ± 0.94	30.59 ± 0.38	0.0425∗
HT	7 (14.0)	21 (38.89)	25 (67.57)	164 (74.21)	<0.0001∗
DM	0	0	5 (13.51)	133 (60.18)	<0.0001∗
Smoking	7 (15.91)	24 (44.44)	17 (45.95)	134 (60.63)	<0.0001∗
CAD	0	0	0	189 (85.52)	<0.0001∗
MI	0	0	0	33 (14.93)	<0.0001∗
Family Hx. CAD	3 (6.0)	17 (31.48)	18 (48.65)	74 (33.48)	0.0001∗
Stroke/TIA	0	0	0	7 (1.93)	0.2075
HF	1 (2.0)	3 (5.56)	1 (2.70)	26 (11.76)	0.0483∗
COPD	1 (2.0)	2 (3.70)	0	3 (1.36)	0.5381
PCI	0	0	0	125 (56.56)	<0.0001∗
CABG	0	0	0	18 (8.14)	<0.0001∗
LDL	3.18 ± 0.16	2.89 ± 0.14	3.15 ± 0.17	2.95 ± 0.07	0.2410
HDL	1.44 ± 0.05	1.14 ± 0.04∗	1.09 ± 0.05∗	0.99 ± 0.02∗	<0.0001∗
Triglyceride	1.70 ± 0.21	2.24 ± 0.20	2.55 ± 0.24∗	2.51 ± 0.10∗	0.0001∗
T. Cholesterol	4.89 ± 0.18	4.59 ± 0.17	5.06 ± 0.20	4.53 ± 0.08	0.0616
Beta blocker	11 (22.92)	25 (46.30)	21 (56.76)	140 (63.35)	<0.0001∗
ACEi	4 (8.33)	8 (14.81)	4 (21.62)	94 (33.48)	0.0004∗
ARBs	4 (8.33)	8 (14.81)	7 (18.92)	46 (20.81)	0.2031
Statins	9 (18.75)	22 (40.74)	27 (72.97)	203 (91.86)	<0.0001∗
Aspirin	9 (18.0)	15 (27.78)	34 (91.89)	200 (90.5)	<0.0001∗
P2Y12i inhibinhibitor	1 (2.0)	2 (3.70)	0	44 (19.91)	<0.0001∗
Diuretics	4 (8.33)	5 (9.26)	5 (13.51)	50 (22.73)	0.0210∗

Data are presented as mean ± sem for continuous variables and n (%) for categorical variables. BMI: body mass index; HT: hypertension; DM: diabetes mellitus; CAD: coronary artery disease MI: myocardial infarction; TIA: transient ischemic attack; Hx: history; HF: heart failure; COPD: chronic obstructive pulmonary disease; PCI: percutaneous coronary intervention; CABG: coronary artery bypass surgery; LDL: low density low protein; HDL: high density lipoprotein; T. Cholesterol: total cholesterol; ACEi: angiotensin converting enzyme inhibitor; ARB: angiotensin receptor blocker; P2Y12 inhibitors: clopidogrel, prasugrel, or ticagrelor. Unit for serum lipids is mmol/L. ∗ indicates presence of significant differences between study groups compared to control using ANOVA or Kruskal Wallis tests for continuous variables and Chi-square test for categorical variables (*p* < 0.05).

Among patients with established clinical ASCVD, 189 patients had CAD≥50% stenosis, and 7 had stroke/TIA. About 50% of patients were smokers. Most of patients had significant co-morbidities; HT, DM, and HF were present in 59.94%, 38.12%, and 8.56% of patients, respectively.

Serum LDL and total cholesterols were not different among study groups, however, levels of TG and HDL were different (Table 1).

A significant correlation between baseline monocytes percentage with ASCVD risk status was found (*p* = 0.0035, Table 1). The risk of ASCVD status was also associated with increased neutrophil/lymphocytes ratio (*p* = 0.037, Table 1).

Most of study patients were on medications to control blood pressure and to prevent ACS and death. About 72.5% of patients were on statins, 71.2% on aspirin, 54.7% on beta-blockers, 43.9% on angiotensin-converting enzyme inhibitor (ACEi)/angiotensin receptor blocker (ARB), and 17.8% were on diuretics.

### 3.2 Association of plasma Lp(a) and t-PA with ASCVD risk status

**Figure 1:**
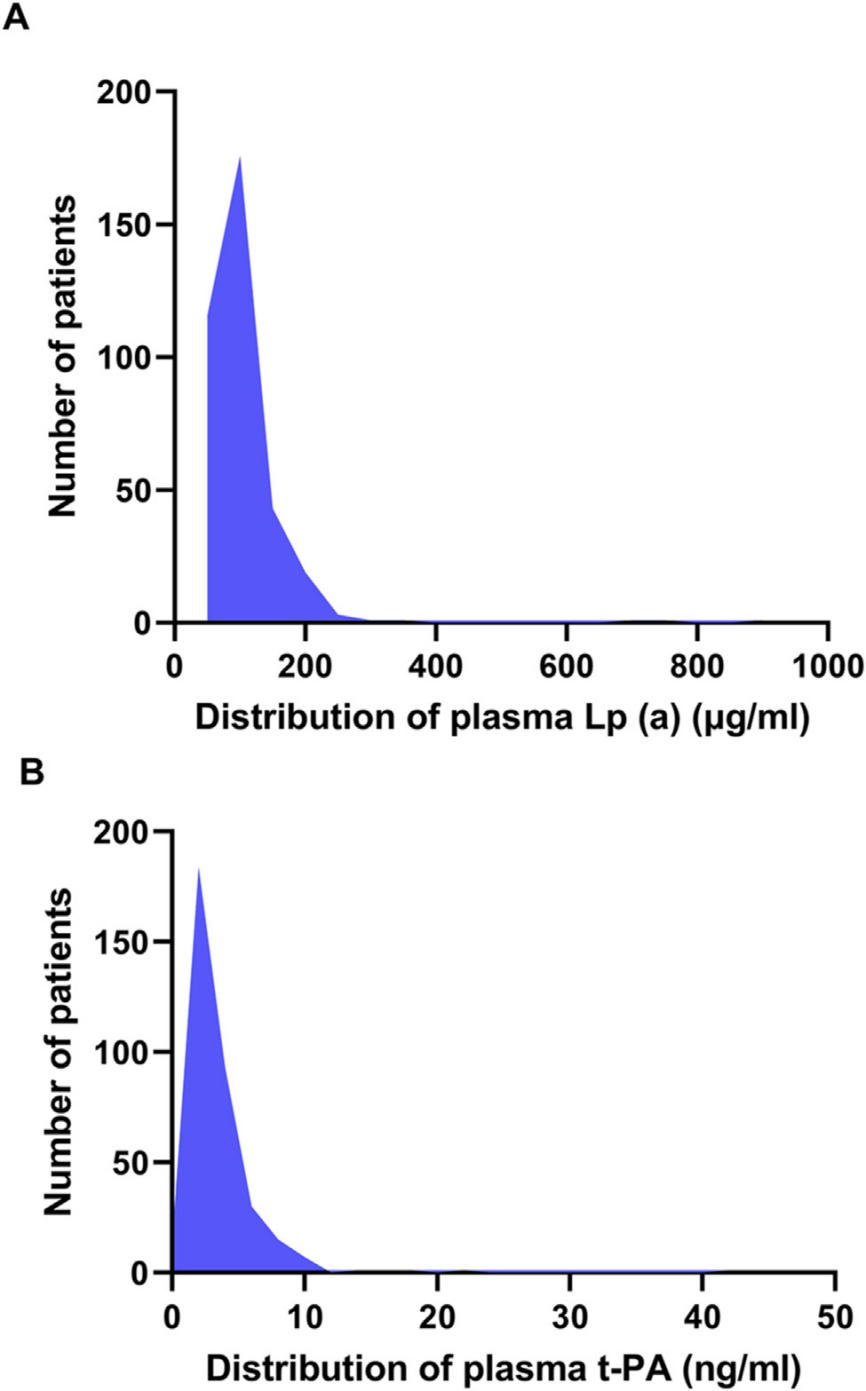
Distribution of plasma Lp(a) and t-PA in the study population. Plasma lipoprotein a (Lp(a), A) and tissue plasminogen activator (t-PA, B) in Jordanian subpopulation, n = 362.

**Figure 2:**
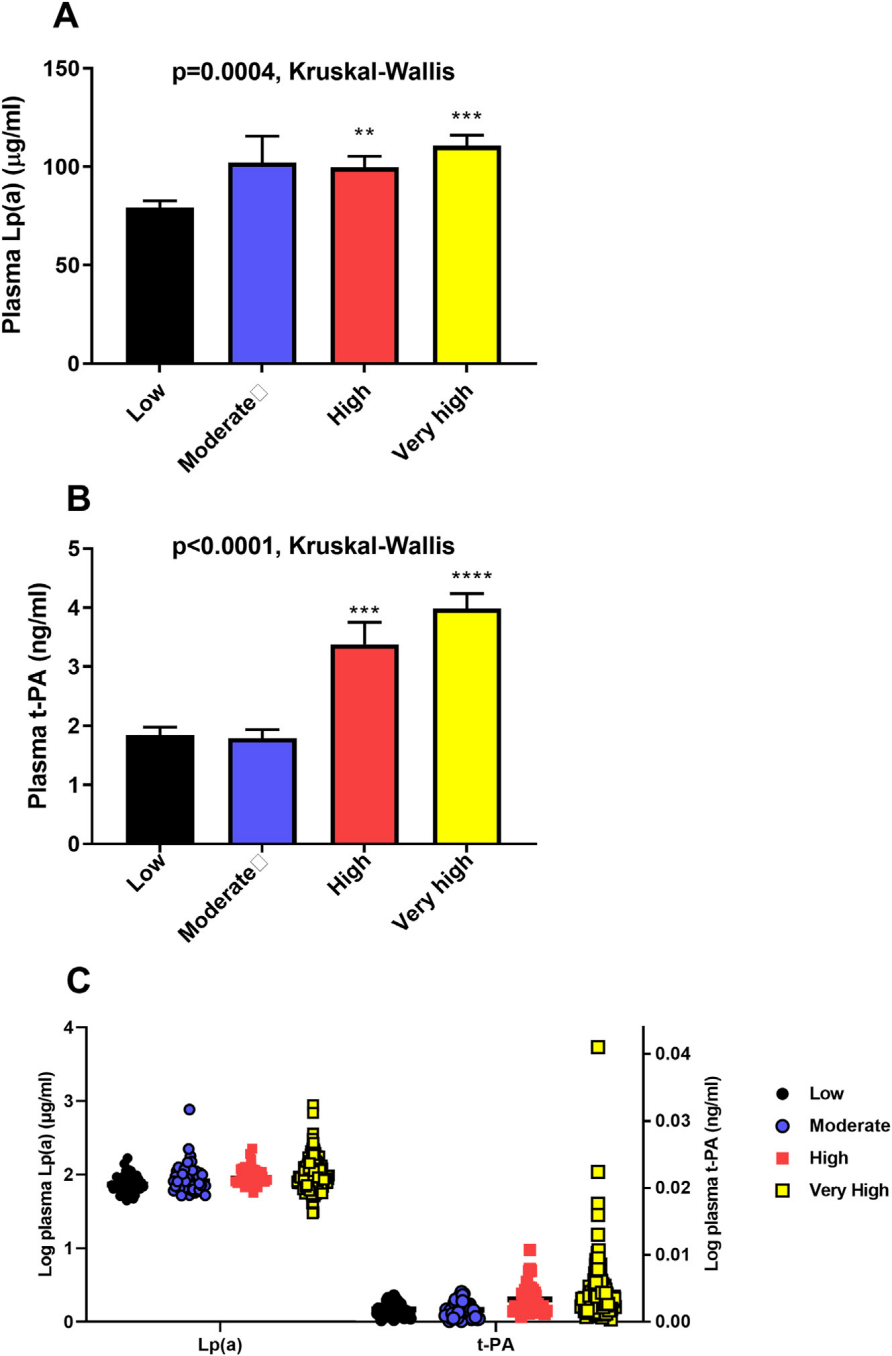
Association of plasma Lp(a) and t-PA levels with ASCVD risk status. Baseline plasma lipoprotein a (Lp(a), A) and tissue plasminogen activator (t-PA, B) in patients with low risk of atherosclerosis cardiovascular disease (ASCVD), moderate risk, high risk, and very high risk. ∗∗*p* < 0.01 and ∗∗∗∗<0.0001 vs. Low. C is a scatter plot for log transformation of plasma Lp(a) and t-PA per ASCVD risk groups.

Figure 1 shows the distribution of plasma Lp(a) and t-PA in our study population (Figure 1 A-B). The distributions are skewed to the right with a tail toward the highest levels. Plasma Lp(a) and t-PA levels were assessed as predictors of ASCVD risk status (Figure 2). Patients with very high and high risk had higher Lp(a) levels than patients with low risk (*p* = 0.0004, Kruskal-Wallis test). No differences in Lp(a) levels were detected between low and moderate risk patients, Figure 2 (A). Similarly, plasma t-PA level was higher in patients with very high and high risk than patients with low risk (*p* < 0.0001, Kruskal-Wallis test), Figure 2 (B). A scatter plot showing log transformation of plasma Lp(a) and t-PA per ASCVD risk is shown in Figure 2 (C). To test whether these associations are modified by LDL, we have stratified LDL levels into three categories; desirable (<2.59 mmol/L), above desirable (2.59–4.11 mmol/L) and high (>4.11 mmol/L). Plasma Lp(a) was significantly associated with increased risk of ASCVD in the above desirable and high LDL categories, but this trend did not reach statistical significance in the desirable LDL category (p = 0.16). On the other hand, Plasma t-PA was significantly associated with increased ASCVD risk regardless or LDL categories (p = 0.003, <0.0001, and 0.0006 for desirable, above desirable and high categories; respectively).

By univariate analysis, statin use was associated with increased levels of plasma Lp(a) (87.4 ± 7.5 vs.110.5 ± 4.6, p = 0.0133). However, by multivariate analysis adjusting for ASCVD risk, statins use was not associated with increased plasma Lp(a) (p = 0.11).

### 3.3 Association of plasma Lp(a) and t-PA with CAD status

**Figure 3:**
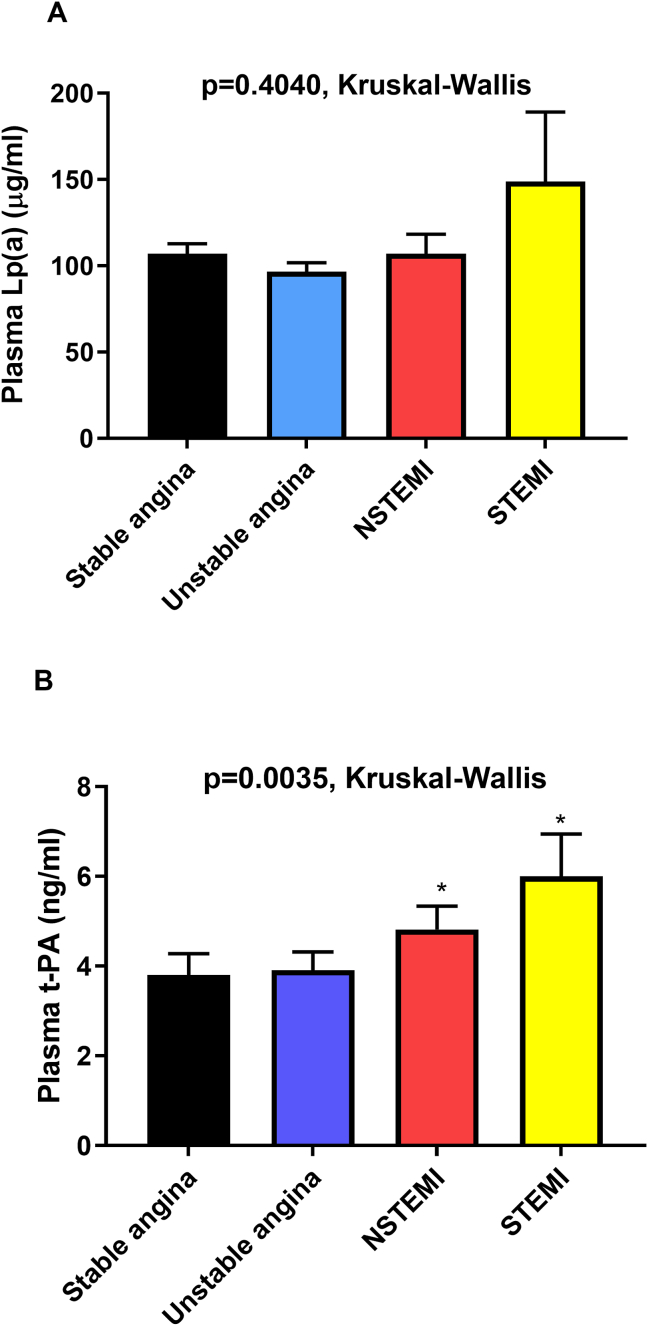
Association of plasma Lp(a) and t-PA with CAD status. Baseline levels of Lp(a) (A) and t-PA (B) in patients with stable angina, unstable angina, non-ST segment elevated myocardial infarction (NSTEMI) and ST-elevated MI (STEMI). ∗*p* < 0.05 and ∗∗*p* < 0.01 vs. stable angina.

Patients with CAD may present with stable angina, unstable angina, non-ST segment elevated MI (NSTEMI), or ST segment elevated MI (STEMI). We assessed plasma Lp(a) and t-PA as a function of CAD status and found no association between Lp(a) and CAD status (*p* = 0.4040, Kruskal-Wallis test), Figure 3 (A). However, plasma t-PA levels were significantly associated with CAD status (*p* = 0.0035, Kruskal-Wallis test). The levels of t-PA were higher in MI patients (NSTEMI and STEMI) relative to stable angina patients, Figure 3 (B). Plasma t-PA level was not different between patients with stable or unstable angina.

### 3.4 Association of plasma Lp(a) and t-PA with coronary intervention

**Figure 4:**
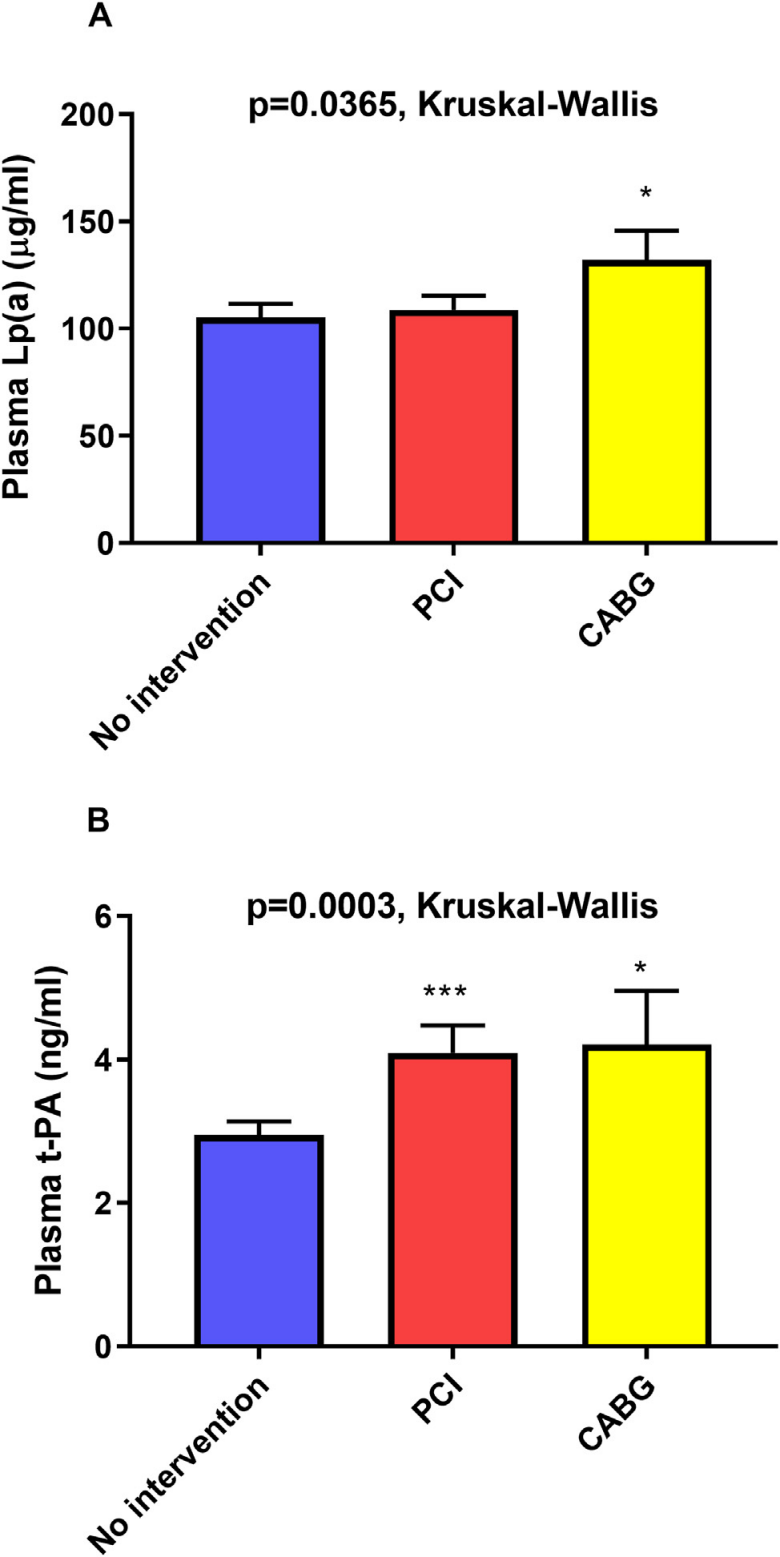
Association of plasma Lp(a) and t-PA with coronary intervention. Baseline levels of Lp(a) (A) and t-PA (B) in patients who underwent percutaneous coronary intervention (PCI), coronary artery bypass surgery (CABG), or underwent no intervention. ∗*p* < 0.05 and ∗∗∗<0.001 vs. no intervention.

We have evaluated plasma Lp(a) and t-PA level as a function of coronary intervention. Patients who presented to the catheterization lab underwent either PCI (stenting, balloon angioplasty, or both), coronary artery bypass surgery (CABG), or no coronary intervention. Plasma Lp(a) level was a predictor of CABG (*p* = 0.0365, Kruskal-Wallis test), Figure 4 (A). Interestingly, plasma t-PA level was significantly higher in patients who underwent PCI or CABG relative to those who had no intervention (*p* = 0.0003, Kruskal-Wallis test), Figure 4 (B). By multivariable nominal analysis adjusting for age, gender, smoking and DM, Log plasma Lp(a) was significantly associated with need for CABG. On the other hand, age, male gender, smoking and DM were associated with need for PCI. Plasma t-PA was marginally associated with need for PCI (p = 0.09, Table 2).

**Table 2:** Multivariate analysis of plasma biomarkers with coronary intervention.

Response = Coronary intervention (no intervention, PCI, and CABG)			
	Estimate (slope, B)	Standard Error	P-value
Age	0.03773	0.01271	0.0030∗
Male gender	0.46342	0. 16550	0.0051∗
smoking	0.14474	0.42697	0.0032∗
Diabetes	0.13151	0.37852	0.0040∗
Log Lipoprotein a	2.6376	1.21330	0.0297∗
Log Tissue plasminogen activator	78.9577	47.5230	0.0966

PCI: percutaneous coronary intervention, CABG: coronary artery bypass surgery, BMI: Body mass index. Data are presented for log odds of CABG/PCI for plasma Lp(a), and log odds of PCI/no intervention for age, gender, smoking, diabetes and plasma t-PA.

### 3.5 Correlation of plasma Lp(a) and t-PA with serum lipids and white blood cells count

To test if plasma Lp(a) and t-PA levels are influenced by serum lipids or inflammatory cells count, we performed a correlation analysis of plasma Lp(a) and t-PA concentrations with serum lipids and white blood cells (WBCs), Table 3. A weak correlation was found between plasma Lp(a) and t-PA suggesting that Lp(a) may contribute little to the expression of t-PA. Plasma Lp(a) levels were not associated with any of serum lipids, monocytes, or neutrophil/lymphocytes ratio. On the other hand, a weak correlation was found between plasma t-PA and serum LDL and triglycerides. Interestingly, plasma t-PA was negatively and significantly associated with plasma HDL. A small correlation was found between plasma t-PA and monocyte percentage, Table 3.

**Table 3:** Correlation of Lp(a) and t-PA with serum lipids and WBCs.

Variable	Lp(a)		t-PA	
	P value	Spearman r	P value	Spearman r
Lp(a)	-	-	0.0431	0.1064
t-PA	0.0431	0.1064	-	-
LDL	0.0907	0.0917	0.0383	0.1122
HDL	0.8978	0.0068	<0.0001	-0.2382
Triglycerides	0.5564	0.0314	0.0253	0.1191
Total cholesterol	0.2319	0.0637	0.1043	0.0864
Monocytes (%)	0.5565	-0.0315	0.0035	0.1559
Neutrophil/Lymphocyte Ratio	0.2276	0.0649	0.1685	0.0741

Data represent the correlation of lipoprotein A (Lp(a)) and tissue plasminogen activator (t-PA) with serum lipids and white blood cells. LDL: low density lipoprotein, HDL: high density lipoprotein. Correlation is spearman correlation.

### 3.6 Multivariate analysis of plasma biomarkers with risk of ASCVD

Multivariate analysis was performed in order to evaluate the association of plasma Lp(a) and t-PA with increased risk of ASCVD adjusting for other factors that may increase the risk of ASCVD (Table 4).

**Table 4:** Multivariate analysis of plasma biomarkers with risk of ASCVD.

Response = Atherosclerotic cardiovascular diseases risk status (ASCVD)				
	Estimate (slope, B)	Standard Error	P-value	Beta Standardized coefficient
BMI	0.01432	0.00981	0.1452	1.46
Log Lipoprotein a	1.08061	0.30326	0.0004∗	3.56
Log Tissue plasminogen activator	135.03106	21.9503	<0.0001∗	6.15
Serum Triglycerides	0.08977	0.03729	0.0166∗	2.41

BMI: Body mass index. Beta (B) = slope/standard error. ASCVD was coded as: low = 1, moderate = 2, high = 3, very high = 4.

Because plasma levels of Lp(a) and t-PA were not normally distributed, a log transformation was used to normalize the data. Multivariate analysis adjusted for triglycerides levels and body mass index (BMI) suggested that both log plasma t-PA and log Lp(a) are significantly and independently associated with increased ASCVD risk. The use of aspirin, statins, ACEis, beta-blockers, and P2Y12 inhibitors was associated with increased ASCVD risk by univariate and multivariate analysis. However, as most of patients with atherosclerosis were on these medications, they were not included in the model to prevent collinearity and underestimation of the effects of other variables with risk of ASCVD. By step-wise analysis, heart failure (HF), serum LDL or LDL categories, and neutrophil/lymphocytes ratio were not associated with ASCVD risk and were excluded from the model. As age, gender, smoking, DM, and HDL level were used as risk factors to stratify patients into ASCVD groups, they were not included in the model to prevent collinearity. In addition to log plasma Lp(a) and t-PA, serum TGs levels were also associated with increased ASCVD risk.

## 4 Discussion

Atherosclerotic cardiovascular diseases contribute significantly to the development of MI, HF, and mortality. Dyslipidemia and endothelial dysfunction are key factors for development of ASCVD. The aim of the present study was to assess plasma Lp(a) and t-PA levels in patients with low to very high risk of ASCVD and their usefulness to predict the need for coronary revascularization. We have adopted the NLA and the AHA/ACC guidelines for risk classification of ASCVD {Grundy, 2019 #38; Jacobson, 2014 #19}.Our study documented elevated levels of plasma Lp(a) and t-PA in patients with increased risk of ASCVD independent of serum LDL. Plasma t-PA levels were associated with CAD status and MI. Both plasma t-PA and Lp(a) were correlated with need for coronary intervention by PCI or CABG. Serum HDL and TGs, but not LDL, were also associated with increased ASCVD risk.

### 4.1 Plasma level of Lp(a)

Plasma Lp(a) levels are highly inherited and stable over time regardless of underlying risk factors or dietary habits [9]. The distribution of plasma Lp(a) in Jordanian subpopulation is similar to previously reported distribution in the general population. The distribution of plasma Lp(a) was skewed to the right with a tail towards the highest levels indicating that genetics largely determine their levels [14]. Population based studies with tens of thousands of patients have clearly shown that genetics can predict both Lp(a) levels, and that Lp(a) levels are causally associated with increased CVD risk independent of LDL levels [9]. In large randomized studies, genetically elevated Lp(a) was associated with increased risk of MI [21]. Interestingly, in a recent study [22], elevated Lp(a) was associated with an increased risk of CAD in the absence of premature family history of CAD, indicating that Lp(a) assessment may be also beneficial in predicting CAD risk in patients without a family history. Thus, current studies are focused on reducing plasma Lp(a) levels by the use of targeted therapies. The use of antisense oligonucleotides was associated with potent reduction of plasma Lp(a) and reduced monocytes’ inflammation in patients with high levels of Lp(a). On the other hand, modest reduction of Lp(a) using subtilisin/kexin type 9 antibody (PCSK9 AB) did not have similar effect [23]. The reduction in CVDs risk is proportional to the absolute reduction in Lp(a) concentration. Large decrease in Lp(a) concentration of about 100 mg/dL may be needed to result in a clinically significant reduction in the risk of CVDs [9]. Furthermore, niacin reduces plasma Lp(a) levels in a dose-dependent manner in addition to its effects on LDL, triglycerides, and HDL cholesterols. Niacin has been shown to reduce coronary artery events, stroke and and cardiovascular events [24].

An elevated level of Lp(a) was documented to predict MI, CAD, and ischemic stroke [25, 26, 27]. Higher Lp(a) level is associated with a higher risk of MI independent of other risk factors [28]. In a large meta analysis, Lp(a) was independently associated with CAD and stroke [29]. Previous studies assessed the usefulness of Lp(a) levels to predict atherosclerotic events; however, it is unknown if Lp(a) levels are increased in patients without underlying atherosclerosis but with risk to develop CAD or MI. In the present study, we evaluated plasma Lp(a) in patients with low, moderate, and high risk of ASCVD who do not have underlying atherosclerosis in addition to those with established ASCVD. We found that Lp(a) level was increased in patients with very high and high risk patients relative to moderate and low risk patients. This association was independent of plasma lipids or presence of other diseases, suggesting that Lp(a) is significantly and independently associated with increased ASCVD risk even in patients without underlying CAD. These data indicate that high Lp(a) may predict or promote the development of atherosclerosis. Both LDL and Lp(a) are lipid particles with similar structure and apolipoproteins, however, we did not find a correlation between Lp(a) level and serum lipids levels, particularly LDL. The association of plasma Lp(a) with ASCVD risk was not confounded by serum LDL. This is consistent with previous report documenting that plasma Lp(a) is associated with atherosclerosis and CAD risk independent of LDL levels [9]. Although plasma Lp(a) was significantly associated with ASCVD risk in the high and above desirable LDL categories, this trend was marginally significant for the desirable range suggesting a potential interaction. Future studies with higher sample size are needed to test if LDL modifies the Lp(a) risk of ASCVD at higher LDL concentrations.

Management of CAD may require coronary revascularization by PCI or CABG. Patients with multi-vessel disease may require CABG. Interestingly, we found an association between Lp(a) levels and coronary interventions; patients who underwent CABG had higher Lp(a) level than other patients, suggesting that Lp(a) levels can predict the severity of coronary lesions in CAD patients. Nevertheless, we did not find a correlation between plasma Lp(a) and CAD status. Only four patients who underwent CABG had MI. As plasma Lp(a) levels are stable and genetically determined, they were not elevated acutely in our MI patients relative to those with angina [9, 22], indicating that the high Lp(a) genetic level could predict the need for CABG.

The neutrophil to lymphocyte ratio is a marker of inflammation and a predictor of MI [30]. Lp(a) has a role in the chemotaxis of monocytes, which plays a major role in atherosclerosis pathogenesis [31]. However, in our study, we did not find an association between Lp(a) levels and monocytes percentage or neutrophils/lymphocytes ratio.

Although neutrophil/lymphocytes ratio was positively associated with ASCVD risk by univariate analysis, this association was not found by multivariate analysis. The absence of an association may be due to the small sample size of patients with available WBC counts. Future studies with a larger sample size may reveal this association.

### 4.2 Plasma level of t-PA

The t-PA is responsible for the conversion of plasminogen to its active form, plasmin, that subsequently dissolves the blood clot and causes fibrinolysis [8]. An association between t-PA levels and CAD risk has been previously found [32, 33]. High baseline level of t-PA was associated with three to four times higher risk of MI and stroke in patients with no prior cardiovascular disease [16]. Our study documented an elevation of t-PA level in patients with very high ASCVD risk and high risk patients who do not have underlying atherosclerosis independent of plasma Lp(a), LDL, or other factors, suggesting the important role of t-PA as a predictor of future ASCVD development.

The structure of Lp(a) is similar to t-PA and it could compete with plasminogen for its binding site [6, 7] leading to reduced fibrinolysis and increased thrombosis risk. Intriguingly, it was proposed that the association of high t-PA with CAD is a result of coronary endothelial injury and subsequent release of t-PA and other associated inflammatory factors. The t-PA may also promote plaque rupture and development of ACS [32]. Interestingly, we found that t-PA level was higher in STEMI and non-STEMI patients than angina patients, suggesting the role of t-PA levels in plaque instability, rupture, platelet aggregation and development of acute MI [34]. It has been found that t-PA has an important role in destabilizing the fibrous cap of atheromatous plaque [35] by initiating proteolysis of the matrix and plaque degradation leading to coronary plaque rupture [36, 37].

Our study is the first to show a strong association between t-PA level and the need for coronary interventions by univariate analysis, where patients who underwent CABG or PCI had higher t-PA levels than those who had no interventions, suggesting that t-PA levels can also assess the severity of atherosclerosis disease and predict the need for coronary intervention by PCI or CABG. However, by multivariate logistic analysis adjusting for other predictors such as smoking, age, and diabetes, the association was marginally significant. Future studies with higher sample size are needed to confirm this association.

The t-PA has a role in the activation of matrix metaloproteases, leading to modification of extracellular matrix composition and macrophage migration [38]. We detected a small correlation between plasma t-PA and monocytes percentage, which may support the t-PA role in the recruitment and activation of inflammatory cells. A weak correlation was also found between plasma t-PA and serum LDL and TGs. A previous study found a positive correlation between t-PA and serum TGs [39]. Interestingly, plasma t-PA was negatively and significantly associated with serum HDL, suggesting that the increase in t-PA is associated with reduction of HDL levels in patients. This finding may also suggest that elevated HDL may play a role in preventing plaque rupture by protecting tissue from t-PA proteolytic effect [40]. Similar to our results, a positive and negative correlation of t-PA antigen with TGs and HDL was found, respectively [41].

### 4.3 Serum lipids and ASCVD risk

Serum LDL is known to be a predictor of CAD and a primary target of therapy in patients [42]. Serum LDL was not associated with ASCVD risk and was similar among our study groups, suggesting that LDL does not predict risk status of ASCVD, and CAD/MI can occur at any level even when LDL is not elevated. It was demonstrated that LDL, even at the normal level, is independently associated with atherosclerosis presence and extent [43]. Interestingly, low levels of LDL (<70 mg/dL or <100 mg/dL) were not associated with decreased prevalence of CAD or with reduced severity [44]. In the present study, Lp(a) and t-PA did not show a marked correlation with LDL and their association with ASCVD risk was not confounded or modified by LDL levels or categories.

An inverse relationship between serum HDL level and CAD is well known [42]. HDL was strongly associated with ASCVD risk but was not included in the model because it was already included in ASCVD risk calculation. Interestingly, we documented that serum TG levels are associated with increased ASCVD independent of Lp(a) and t-PA levels. It was found that high TGs level was associated with increased MI risk and death [45]. It has always been known that LDL rather than TG is involved in atherosclerotic plaque formation [46]. However, recent genetic studies provide robust evidence that TGs and TG rich lipoproteins are in the causal pathway for atherosclerosis [47], indicating that assessment of patients risk of atherosclerosis should also focus on triglyceride levels.

### 4.4 Study strengths

This is the first study published in Jordanian population to asses plasma Lp(a) and t-PA with increased risk of ASCVD. Our study revealed that the distribution of plasma Lp(a) is similar to the general population [14]. Although many studies assessed the levels of Lp (a) in CAD patients, our study provides assessment of their levels using a cumulative risk classification adopted by the national lipid association [20] including patients with and without CAD with various risks to develop ASCVD. Our study shows that plasma Lp(a) levels are elevated not only in CAD patients (very high risk patients), but also in those without CAD who are at high risk to develop CAD, highlighting that therapy and preventable measures should be used at this stage to reduce risk of atherosclerosis development. In addition, our study is the first to examine the significance of Lp(a) and t-PA in predicting the need for coronary intervention by PCI or CABG. Further more, the study investigated if there is a link or correlation of plasma Lp(a) with t-PA.

### 4.5 Study limitations

This study is limited by the relatively small number of included patients and the cross-sectional design of the study at a single center in Jordan. However, our center is the major center in the North of Jordan that performs catheterization. Given the molecular similarity of LDL and Lp(a), current LDL assays (inappropriately) include Lp(a) levels in the measurement which might have affected the interpretation of LDL contribution to ASCVD [48].

## 5 Conclusions

Our study suggests that both plasma Lp(a) and t-PA are associated with increased ASCVD risk independent of LDL levels, and were increased in very high risk patients and high risk patients who do not have underlying atherosclerosis, suggesting their roles as predictors of atherosclerosis development and progression. Both Lp(a) and t-PA levels were increased in patients who underwent coronary revascularization, indicating their association with severity of coronary lesions and their possible role to select patients who may benefit from coronary revascularization. The correlation between high t-PA levels with MI supports the role of t-PA in plaque rupture and MI development.

## Declarations

### Author contribution statement

Fadia Mayyas: Conceived and designed the experiments; Analyzed and interpreted the data; Wrote the paper.

Eman Bani Omar: Performed the experiments; Analyzed and interpreted the data; Wrote the paper.

### Funding statement

Dr Fadia Mayyas was supported by this work was funded by a grant from Deanship of Scientific Research at Jordan University of Science and Technology [246/2020].

### Data availability statement

Data will be made available on request.

### Declaration of interests statement

The authors declare no conflict of interest.

### Additional information

No additional information is available for this paper.
